# The combined effect of perception of pay fairness and institutional transparency on physicians’ work enthusiasm: a mixed method study from China

**DOI:** 10.3389/fpubh.2025.1555684

**Published:** 2025-05-30

**Authors:** Ning Hu, Jing Ma, Wei Wei, Xiaoying Jiang, Aiping Zhu, Chunyu Zhang

**Affiliations:** ^1^School of Management, Beijing University of Chinese Medicine, Beijing, China; ^2^Emergency Department, People's Hospital of Ningxia Hui Autonomous Region, Yinchuan, China; ^3^Youth League Committee, Linyi People's Hospital, Linyi, China; ^4^Department of Science, Beijing Chest Hospital Affiliated to Capital Medical University, Beijing, China; ^5^Hospital Office, China-Japan Friendship Hospital, Beijing, China; ^6^Department of Human Resources, China-Japan Friendship Hospital, Beijing, China

**Keywords:** physician, moderating role, institutional transparency, pay fairness, work enthusiasm, structural equation modeling, mixed study

## Abstract

**Introduction:**

Physician salary structures in Chinese public hospitals are crucial for motivating healthcare providers. Based on Expectancy Theory, this study aims to provide an evidence-based foundation for hospital managers in salary system design through investigating the combined effect of perceptions of pay fairness and institutional transparency on Chinese physicians’ work enthusiasm, with a focus on the moderating effect of institutional transparency.

**Methods:**

A qualitative study was conducted from December 2023 to February 2024 to initially explore the relationships of perception of pay fairness, institutional transparency, and work enthusiasm. Subsequently, a quantitative study was conducted between April and May 2024 to examine the combined interaction of these factors. Physicians from Beijing, Haikou, Linyi, Nanjing, and Yinchuan were recruited to participate in the study.

**Results:**

The qualitative analyses showed that most physicians knew the determinants of salary but were not familiar with the details. The monthly salary gaps acceptable by most physicians featured time allocation ranging from 0.5 to 1.0, or less than ¥10,000 ($1,378). The structural results of the equation modeling showed that the perception of pay fairness had a significant and positive impact on work enthusiasm (*β* = 0.454, *p* < 0.001), and institutional transparency promoted the association between the perception of pay fairness and work enthusiasm (*β* = 0.229, *p* = 0.003).

**Conclusion:**

Reasonable salary gaps and awareness of the scheme are required to improve physicians’ enthusiasm for their work.

## 1 Introduction

Within the clinical workflow, physicians serve as the primary decision-makers, exercising critical judgment in both diagnostic strategy development and treatment execution. Therefore, their work enthusiasm significantly affects the efficiency and quality of medical services, and ultimately impacts patient satisfaction (1, 2).

Research indicates that salary is a key motivator for employees to actively engage in their work (3–5), and studies have demonstrated that both the absolute value of salary and the perception of pay fairness and institutional transparency are essential for activating motivation (6, 7). Physicians’ salaries in Chinese public hospitals consist of three components: basic salaries, bonuses, and benefits. The bonuses, which are determined by performance, account for more than half the total salary, and there is a salary gap due to the unique regulations of hospitals or departments (8). In China, distributive justice and transparency are particularly important, and Chinese physicians are increasingly concerned about how they are paid.

Previous studies have examined the separate, but not the combined impacts of the perception of pay fairness and institutional transparency on work enthusiasm (9, 10). Vroom’s Expectancy Theory posits that salary serves as a powerful incentive for boosting work enthusiasm when it aligns with expectations (11). Salary expectations include both equity in reward-for-effort and parity with peers performing comparable duties. When the salary scheme is kept confidential, employees tend to assume that others earn more than they do for doing similar work, and the sense of injustice increases, resulting in dissatisfaction.

**Figure 1:**
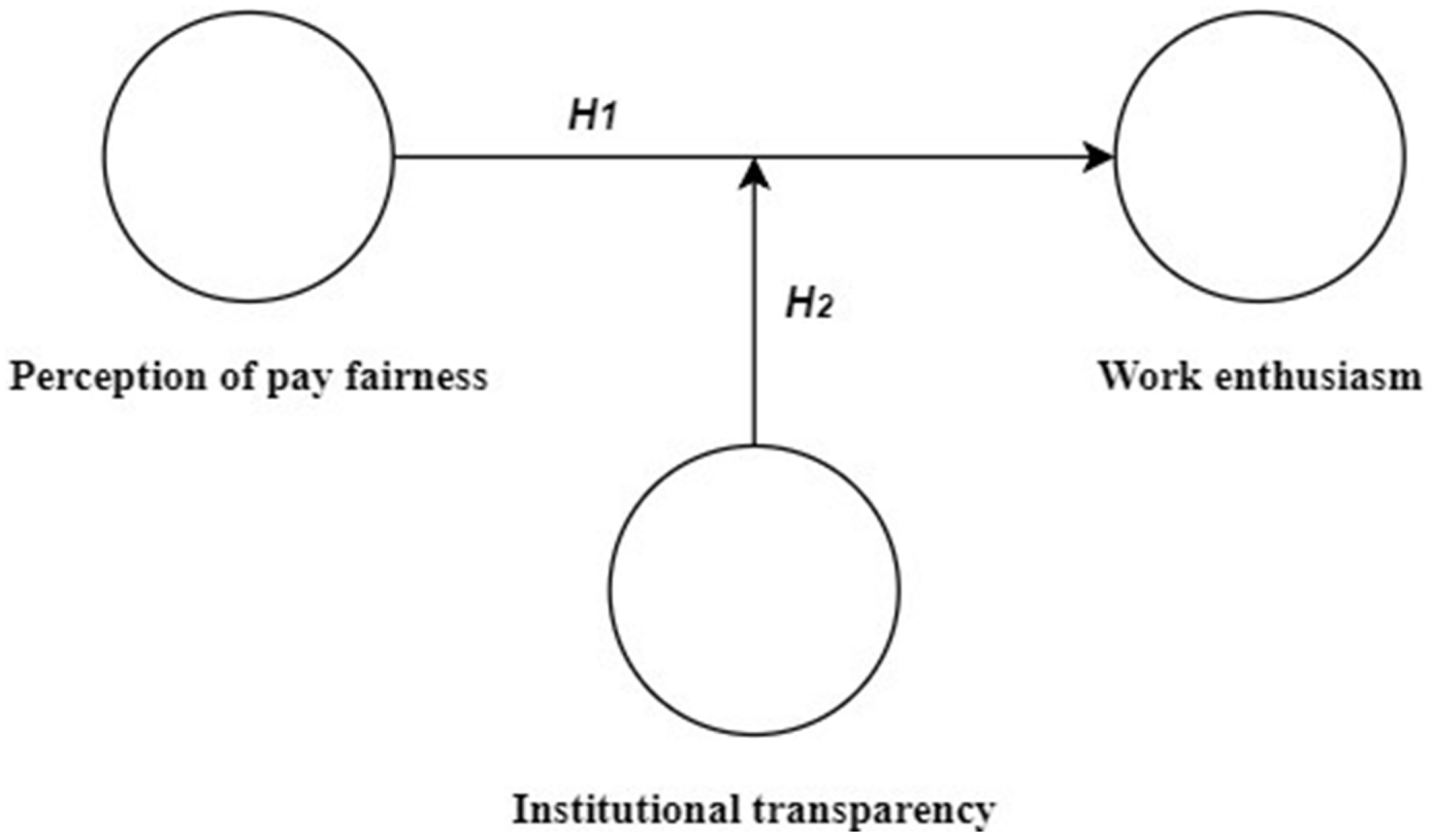
The conceptual model based on study hypotheses.

Our research is based on Expectation Theory. First, through in-depth interviews, we explore whether institutional transparency and perception of pay fairness affect work enthusiasm in China. We then construct a structural equation model to quantify the influence of perception of pay fairness on work enthusiasm and the moderating effect of institutional transparency (Figure 1). These evidence-based insights offer actionable references for hospital managers to optimize remuneration frameworks.

## 2 Literature review and research hypotheses

### 2.1 Literature review

#### 2.1.1 Perception of pay fairness and work enthusiasm

Fair pay can enhance employees’ job satisfaction and work enthusiasm, while unfair pay may lead to employees reducing work involvement and even increasing turnover intention. Several studies have confirmed that there is a significant positive correlation between pay fairness and turnover intention. When employees believe that their pay matches their efforts and performance, they feel it is fair and are more willing to work (12–14). Perception of pay fairness is also critical in healthcare. When physicians believe that their pay is fair and reasonable, they are more actively engaged in clinical work and provide high-quality services to patients (10).

#### 2.1.2 Institutional transparency and work enthusiasm

Studies across various business sectors underscore the importance of institutional transparency in increasing job satisfaction and elevating enthusiasm for work (15). Transparent institutional decision-making and salary distribution systems can enhance employees’ trust in the organization, reduce misunderstanding and the sense of injustice caused by information asymmetry, and make employees more willing to contribute to the realization of organizational goals (16). Physicians, like most employees around the world, desire to be better informed about their salaries. Previous research found that physicians who perceived institutional transparency were more competitive and honorable, less likely to burn out, and more ready to devote themselves to healthcare services (17).

#### 2.1.3 Perception of pay fairness and institutional transparency

Salary transparency has been implemented in various ways around the world as a strategy by firms and policymakers to reduce the gender pay gap. Lyons and Zhang examined whether salary transparency influences gender pay inequality in the context of Canadian universities and found that salary disclosure lowered the cost of monitoring the gender pay gap and enhanced employees’ perception of pay fairness (18).

In conclusion, both perception of pay fairness and institutional transparency have an important impact on physicians’ work enthusiasm, and institutional transparency regulates the perception of pay fairness. Based on the Expectation Theory, this study further explores the relationship among perception of pay fairness, institutional transparency, and physicians’ work enthusiasm in the context of Chinese hospitals, to provide a theoretical basis and practical guidance for improving physicians’ work enthusiasm and improving the quality and efficiency of medical services provided in China.

### 2.2 Research hypotheses

Based on the above analysis and Expectancy Theory, we proposed the following hypotheses (Figure 1):

*H1*: Physicians’ perception of pay fairness have a direct impact on their work enthusiasm.

*H2*: Institutional transparency moderates the positive correlation between perception of pay fairness and work enthusiasm. When institutional transparency is high, the relationship between perceived pay fairness and work enthusiasm is strengthened.

## 3 Methods

This study used a combination of qualitative and quantitative research methods. Qualitative analysis was used to explore whether the association exists, and quantitative analysis was used to verify whether the association is statistically significant. Structural equation modeling (SEM) was used to test the effect of perception of pay fairness on physicians’ work enthusiasm, and the moderating effect of institutional transparency on perception of pay fairness and work enthusiasm. SEM is a multivariate statistical analysis technique that combines multiple regression and factor analysis methods to automatically evaluate a series of interrelated causal relationships. SEM is similar to multiple regression in its use but is more suitable for a variety of complex situations and has been applied in the field of health economics (19). Therefore, SEM was used in this study to estimate the effects of perception of pay fairness and institutional transparency on physicians’ work enthusiasm. All analyses were performed using SPSS 24.0 and Amos 29.0 software packages.

### 3.1 Study design

**Figure 2:**
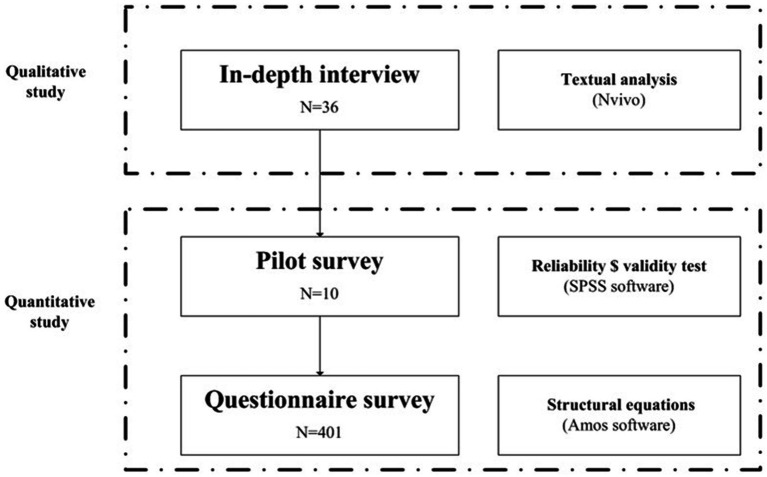
Study design.

As shown in Figure 2, before the quantitative study, face-to-face interviews were conducted with the physicians (*n* = 36) to explore how they perceived institutional transparency, their opinions about the salary gap, and the impact of the two items on work enthusiasm. Interviews took place from 12 December 2023 to 29 February 2024. The first stage of the quantitative study was a pilot survey with a small sample size (*n* = 10) to revise the questionnaire. In the second stage, we conducted a cross-sectional survey in China from 16 April to 17 May 2024. Hospitals in Beijing, Haikou, Linyi, Nanjing, and Yinchuan were selected to participate, as these cities are from regions with different levels of economic development in China, making the sample representative. Our research team recruited physicians from the selected hospitals using convenience sampling, helped participants scan the QR codes, and answered questions to assist participants in completing the questionnaire smoothly. We determined that a sample of 300 physicians was sufficient to test our hypotheses using SEM (20, 21). We continued recruiting 101 physicians through purposive sampling, balancing sex and age to ensure the representativeness of the sample and to reach a valid sample size. In total, 401 physicians responded to the survey. Among them, 59 questionnaires were excluded because of unreasonable response durations or logistic errors. Ultimately, 342 questionnaires and 36 interviews were included in the final analysis.

### 3.2 Measurements

Semi-structured interviews were conducted using the following three questions: (1) “How much do you know about the salary scheme?” (2) “What salary gap do you regard as reasonable?” and (3) “Would you change your work behavior if you were to perceive salary unfairness?”

The questionnaire survey comprised four parts: demographics, institutional transparency, perception of pay fairness, and work enthusiasm. A five-point Likert scale was used to rate perceptions of institutional transparency, pay fairness, and work enthusiasm, with 1 indicating “strongly disagree” and 5 indicating “strongly agree.” The institutional transparency section consisted of four questions from three aspects: degree, timeliness, and way of learning about the pay structure. The Cronbach’s alpha for the scale was 0.947, demonstrating excellent reliability (Table 1). The section on perception of pay fairness consisted of seven questions on four aspects: external comparisons, internal comparisons, self-comparisons, and satisfaction with the performance allocation scheme. The Cronbach’s alpha for the scale was 0.944, which demonstrated excellent reliability (Table 1).

**Table 1:** Questionnaire structure.

Aspect	Questions
Institutional transparency	1. In my opinion, I am very familiar with the department performance allocation scheme
	2. When I want to know the performance allocation scheme, I can easily find out
	3. I can know the department performance allocation scheme ahead of time
	4. The superior leader took the initiative to share the performance distribution with me
Perception of pay fairness	1. Compared to other positions in the hospital, my salary is reasonable
	2. Compared with physicians in other departments, my salary is reasonable
	3. Compared with the higher ranked physicians within the department, my salary is reasonable
	4. Compared with physicians of similar rank within the department, my salary is reasonable
	5. The salary I received matches my efforts and contributions
	6. The salary I actually received was the same as the salary I expected
	7. I think the performance distribution scheme in the department is fair and equitable
Work enthusiasm	1. I believe that I am fully capable of performing my job
	2. My job has brought me social respect and recognition from patients
	3. I hope to treat more patients, and I do not mind the hardship involved
	4. I would not focus my efforts on areas other than medical work (such as research, lecturing, etc.) to increase my income
	5. I have never had the thoughts of turnover

The work enthusiasm section consisted of five questions on three aspects: job competence, career achievement, and job satisfaction. The Cronbach’s alpha for the scale was 0.769, which demonstrated good reliability (Table 1).

In terms of all dimensions, the Kaiser–Meyer–Olkin (KMO) coefficient was 0.926, Bartlett’s test of sphericity was (*p* < 0.001), and the Cronbach’s alpha coefficient was 0.875, indicating very good reliability and validity.

### 3.3 Statistical analysis

After the audio recordings were transcribed, NVivo 12 (QSR International) was used to code the transcripts. Members of the research team first coded the transcripts independently, then discussed and resolved any discrepancies. SPSS software (version 24.0; IBM) was used to analyze the reliability and validity of the questionnaire. AMOS software (version 29.0; IBM) was used to construct structural equation modeling to test the hypotheses. The factor structures of the scales used were examined using exploratory factor analysis (EFA) and confirmatory factor analysis (CFA). Additionally, the study assessed the moderating effect of institutional transparency on the relationship between perceptions of pay fairness and work enthusiasm. The significance of the indirect effect was based on a 95% confidence interval (CI) obtained through bootstrapping, with 5,000 bootstrap samples.

### 3.4 Ethical considerations

The study protocol was approved by the Ethics Committee of the China-Japan Friendship Hospital (2022-KY-218). Participants were offered one yuan (0.14 US dollars) via digital transfer upon full survey completion to acknowledge their participation time. One yuan was deliberately set below the threshold that might be coercive for physicians. All questions were mandatory. The survey was anonymous, with the answers sent directly to a database to ensure confidentiality. The participating physicians gave informed consent before starting the online questionnaire or interview. Participants could withdraw at any time. There was no patient or public participation in this study.

## 4 Results

### 4.1 The results of the qualitative study

The results of the in-depth interviews indicated that institutional transparency was a concern among physicians, and an unfair salary gap would affect their work enthusiasm. In terms of institutional transparency, only two physicians (one who was the director of a department and the other who was not the leader but was involved in bonus distribution) reported they were familiar with the salary scheme and colleagues’ salaries. The remaining physicians knew the determinants of salary but were not familiar with the details. All physicians believed a more transparent salary scheme would benefit clinic work. This belief was summarized by an associate professor of neurosurgery, who told us the following:

Our salary is determined by academic rank, workload, and efficiency. Due to busy clinic work, I know little about the weight of the determinants, but the more patients or the higher a case mixed index, or shorter average length of stay, the more I earned. I am eager to know the scheme and how much my colleagues earn. The transparency could avoid suspicion and lead to virtuous competition.

The results of the in-depth interviews showed that a salary gap ranging from a time allocation of 0.5 to 1.0, or less than ¥10,000 ($1,378), is considered acceptable by the majority of physicians. An Associate Professor of Obstetrics and a Resident of Gastroenterology Physicians described this as follows:

Although residents undertook numerous tasks, senior physicians were responsible for the risk during the whole treatment. For example, during the COVID-19 pandemic, almost all physicians were infected. It was the senior physicians who still did the operations through coughing and fever. So, I think senior physicians should earn more than junior physicians, and the gap should reach 0.5 to 1 time.

I agree that the senior physician should earn more due to having more responsibility. However, considering I and the attending physician take care of the same patients, the reasonable salary gap is less than 10,000 yuan. In terms of same-level physicians from different departments, the salary gap should also be less than 1,000 yuan to avoid the unfairness among specialties.

When physicians felt that the salary was unfair, most interviewees resorted to other measures to increase their income, such as scientific study, outpatient clinics, and other activities. Despite this, they still treated patients as usual, but avoided caring as much as possible. A professor of endocrinology reported the following:

When my salary is less than other physicians, I will stick to treating patients based on the needs of their disease due to professional morality. However, I will reduce the number of admitted patients and spend more time on other respects to increase my income. For example, I would transfer to scientific research and acquire more research funding.

### 4.2 The results of the quantitative study

#### 4.2.1 Characteristics of participating physicians

The participants were 48.83% male and 51.17% female. The majority of the participants (81.00%) were between 31 and 50 years of age; 41.23% were between 31 and 40, and 39.77% were between 41 and 50 years. Nearly half of the participants (48.54%) held a master’s degree, 29.24% had a bachelor’s degree, and 22.22% had a doctoral degree. Most participants had over 10 years of work experience (70.76%), followed by 11–15 years (28.07%) and 16–20 years (20.76%). The participants’ specialties were primarily in internal medicine or surgery. The monthly salary of participants was mostly between 10,001 and 20,000 yuan (1,397–2,793 US dollars), followed by 6,001–10,000 yuan (838–1,396 US dollars) (Table 2).

**Table 2:** General characteristics of participant physicians.

Characteristic	Categories	Frequency	Percentage
Sex	Male	167	48.83%
	Female	175	51.17%
Age	≤30 yrs	43	12.57%
	31–40 yrs	141	41.23%
	41–50 yrs	136	39.77%
	51–60 yrs	19	5.56%
	≥61 yrs	3	0.88%
Educational level	Bachelor’s degree	100	29.24%
	Master’s degree	166	48.54%
	Doctoral Degree	76	22.22%
Academic title	Resident doctor	65	19.01%
	Attending physician	126	36.84%
	Associate chief physician	128	37.43%
	Chief physician	23	6.73%
Work experience (years)	≤ 1 yr	19	5.56%
	2–5 yrs	46	13.45%
	6–10 yrs	54	15.79%
	11–15 yrs	96	28.07%
	16–20 yrs	71	20.76%
	21–25 yrs	37	10.82%
	≥26 yrs	19	5.56%
Specialty	Surgery	82	23.98%
	Internal medicine	84	24.56%
	Gynecology and obstetrics	12	3.51%
	Department of pediatrics	24	7.02%
	Traditional Chinese medicine	31	9.06%
	General practice	20	5.85%
	Emergency	22	6.43%
	Other	67	19.59%
Monthly salary	≤¥3,000 ($419)	15	4.39%
	¥3,001–6,000 ($419–837)	38	11.11%
	¥6,001–10,000 ($838–1,396)	117	34.21%
	¥10,001–20,000 ($1,397–2,793)	124	36.26%
	¥20,001–30,000 ($2,793–4,190)	33	9.65%
	¥30,001–40,000 ($4,190–5,586)	9	2.63%
	¥40,001–50,000 ($5,586–6,983)	3	0.88%
	≥¥50,000 ($6,983)	3	0.88%

#### 4.2.2 Model fit test and modification

Goodness-of-fit for the model was measured using chi-square/df, goodness-of-fit index (GFI), adjusted goodness-of-fit index (AGFI), normed fit index (NFI), comparative fit index (CFI), incremental fit index (IFI), and root mean square error of approximation (RMSEA). The chi-square/df (2.164), GFI (0.944), AGFI (0.911), NFI (0.970), CFI (0.983), IFI (0.983), and RMSEA (0.058) were within the acceptable ranges, indicating a good model fit.

Convergent validity represents the correlation between different measurement items for the same variable and was measured by factor loadings, average variance extracted (AVE), and composite reliability (CR). Both AVE and CR were found to be greater than the threshold values (22, 23) for all items. The results indicated good convergent validity among the measured items (Table 3).

**Table 3:** Convergent and discriminant validity.

Construct variables	Convergent validity			Discriminant validity		
	*α* > 0.7	AVE > 0.5	CR > 0.7	PF	IT	WE
PF	0.944	0.703	0.904	**0.838**		
IT	0.947	0.829	0.951	0.810	**0.910**	
WE	0.769	0.521	0.765	0.728	0.668	**0.722**

PF, Perception of pay fairness; IT, Institutional transparency; WE, Work enthusiasm.

*α* = Cronbach’s alpha; CR, Composite reliability; AVE, Average variance extracted.

The bold value indicates adequate discriminant validity among the constructs.

Discriminant validity assesses the extent to which constructs are different from each other. It was measured by comparing the square root of the AVE of each construct with the correlation coefficients between variables. When the square of the correlation between the components was less than the predicted dimension AVE, the discriminant validity criterion was fully satisfied (24). The results demonstrated good discriminant validity since the AVE values for all constructs were greater than the squared correlations between constructs (Table 3). To meet the requirements of SEM, modifications were made to the model. Items with factor loadings below the threshold value of 0.60 and squared multiple correlations (SMC) below the threshold value of 0.36 were deleted.

#### 4.2.3 Hypothesis testing

##### 4.2.3.1 The impact of perception of pay fairness

**Figure 3:**
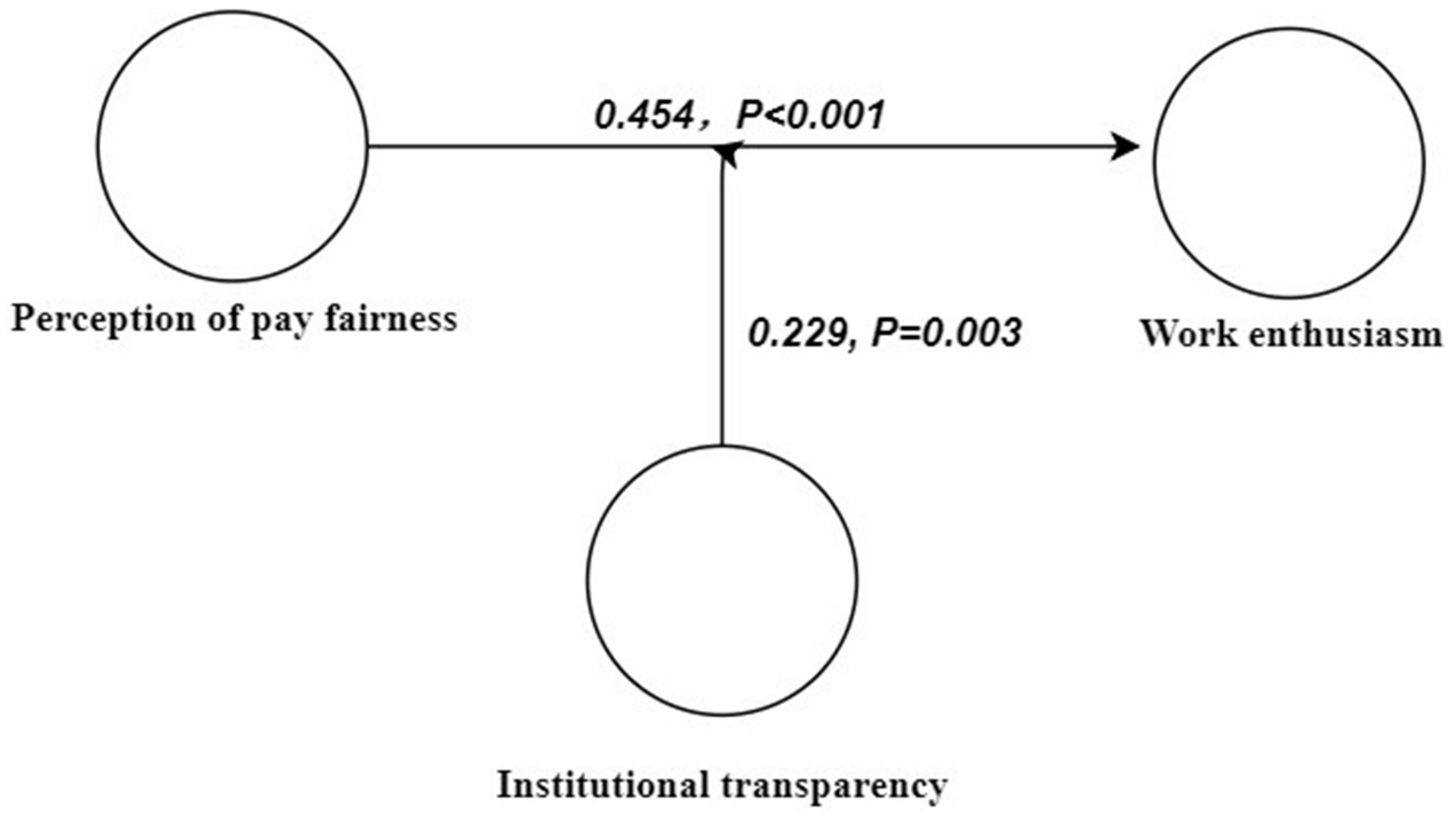
Structural equation modeling of the moderating effects for perception of pay fairness, institutional transparency on work enthusiasm.

The standardized regression coefficient between the perception of pay fairness and work enthusiasm was 0.454, which was statistically significant (*p* < 0.001). This result indicated that perception of pay fairness had a positive and significant impact on work enthusiasm (*β* = 0.454; *t* = 4.304; *p* < 0.001). Therefore, H1 was supported (Table 4; Figure 3).

**Table 4:** The result of the structural equation modeling.

Hypothesis	*β*	SE	*T* value	*p* (Sig)
H1: perception of pay fairness→ work enthusiasm	0.454	0.075	4.304	<0.001
H2: moderating effect → work enthusiasm	0.229	0.041	3.012	0.003

SE is Standard Error.

##### 4.2.3.2 The moderating role of institutional transparency

The results showed that institutional transparency positively moderated the relationship between the perception of pay fairness and work enthusiasm. In the case of high institutional transparency, perception of pay fairness had a stronger positive effect on work enthusiasm (*β* = 0.229; *t* = 3.012; *p* = 0.003). Therefore, H2 was supported (Table 4; Figure 3).

## 5 Discussion

To the best of our knowledge, this study represents the first investigation into the moderating role of institutional transparency in the context of Chinese physicians. By integrating quantitative analysis with insights from in-depth interviews, we offer a comprehensive and detailed examination. The qualitative study found that when physicians perceive their salaries to be unfair, they may become less motivated and seek additional income through non-clinical work. All physicians are eager to understand the performance distribution system and even the incomes of their colleagues. These findings were further confirmed in the quantitative analysis, which demonstrated that perception of pay fairness had a significant direct impact on physicians’ work enthusiasm, and that institutional transparency played a moderating role in increasing the effect of the perception of pay fairness on work enthusiasm.

Our findings strongly support Hypothesis 1, as the perception of pay fairness was found to have a significant and positive effect on physicians’ work enthusiasm. This aligns with existing research in the business domain, which emphasizes that employees’ perception of salary fairness is a critical driver of job satisfaction and work enthusiasm (25, 26). Qualitative interviews revealed that physicians might prioritize non-clinical activities (e.g., research or administrative tasks) over patient care if they perceived their salary as unfair. This behavior reflects a rational response to perceived inequity, as employees seek to balance their efforts and income in the workplace (5). Thus, our study confirms that perception of pay fairness is a key determinant of physicians’ work enthusiasm. Putnam-Farr and Morewedge reported that social comparisons affected salary satisfaction, and employees felt dissatisfied when their salary was below the average level for similar work (27). In essence, satisfaction with salary stems from judgments of fairness, encompassing internal and external, as well as reward-for-effort comparisons. Numerous studies, utilizing both behavioral observations and self-reported data, have underscored individuals’ aversion to unfair treatment, illustrating that unfair compensation triggered decreased brain activity and caused unhappy feelings among individuals (28, 29). Fairly compensating physicians for their work is justified. Pay parity does not imply equal pay, but that a reasonable salary gap elicits significant positive emotions (28), ultimately contributing to a diverse workforce and business success (30). The physicians in our study also reported that a salary gap of 0.5 to 1 times, or less than ¥10,000 ($1,378), between senior and lower academic ranks, and also within the same academic rank, is acceptable. Hospital managers should optimize the salary gap to stimulate physicians’ enthusiasm across all ranks.

We are the first to confirm the hypothesis that institutional transparency moderates the relationship between the perception of pay fairness and work enthusiasm, as demonstrated using SEM. Institutional transparency in salary schemes fosters trust between physicians and hospital managers. When physicians perceive that their institution is open and fair in its salary practices, they are more likely to feel valued and motivated to contribute to their work (31). Institutional transparency affects work enthusiasm by adjusting the perception of pay fairness, because a lack of transparency in a salary scheme tends to create inflated salary expectations, consequently cultivating perceptions of distributive injustice. Institutional transparency reduces ambiguity about salary allocations, thereby minimizing social comparisons and perceptions of inequity. As highlighted in the qualitative interviews, physicians who understood the salary scheme reported less dissatisfaction and were more willing to engage in collaborative work. This finding suggests that transparency amplifies the positive effect of pay fairness on work enthusiasm.

Previous evidence from businesses and a multicenter study in the United States has suggested that institutional transparency can reduce salary inequality (18, 32). However, these studies primarily focused on the direct effects of institutional transparency, without exploring its moderating role in the relationship between perception of pay fairness and work enthusiasm. Our study demonstrates that institutional transparency can enhance the motivational impact of perception of pay fairness.

Our study provides novel insights into how transparency can be leveraged to optimize physicians’ work enthusiasm. This finding is particularly relevant in the Chinese context, where performance-based salaries are common but transparency in allocation schemes remains limited. In China, the majority of physicians’ salaries follow a performance-based model under which all physicians should be offered equal pay for similar work (33). A transparent approach to performance-based salaries grounded in principles of equity is needed; offering more information about performance allocation schemes, performance feedback, and broader explanatory information could help managers establish equal pay for equal work and eventually promote work enthusiasm.

## 6 Conclusion

We used SEM to explore the causal pathways between perception of pay fairness, institutional transparency, and work enthusiasm, based on data derived from in-depth interviews and a questionnaire survey. The results of this study indicate that perception of pay fairness had a direct and significant positive effect on work enthusiasm, and that institutional transparency served as a moderator, amplifying the impact of pay fairness on work enthusiasm. These results contribute to the theoretical understanding of motivation in healthcare settings and offer practical guidance for hospital managers to enhance physicians’ engagement through equitable and transparent compensation practices.

The study also highlights the importance of optimizing salary gaps, increasing transparency in salary distribution, and addressing perceived inequity to foster a more motivated medical workforce. Future research could further explore these dynamics across diverse healthcare systems and cultural contexts, potentially incorporating longitudinal designs and richer qualitative insights to provide a more comprehensive understanding of the factors influencing physicians’ professional engagement.

### 6.1 Limitations

This study focused on the effects of salary on work enthusiasm; however, some non-financial factors may also act as confounding factors, such as opportunities for career advancement and workload, which will be comprehensively considered in future studies. Furthermore, although our research team explained the aim of the study and explained data confidentiality, the relationship among perceived institutional transparency, pay fairness, and work enthusiasm still carries the risk of being under- or overestimated due to the study’s self-report design. Lastly, although our results align with previous research, and the study is the first to establish the moderating role of institutional transparency, further studies with larger samples are needed in future research to validate and generalize our results.

## Data Availability

The raw data supporting the conclusions of this article will be made available by the authors, without undue reservation.
